# Surgical treatment of bronchial asthma by resection of the laryngeal nerve

**DOI:** 10.1186/s12893-015-0093-2

**Published:** 2015-10-08

**Authors:** Ubaidullo Kurbon, Hamza Dodariyon, Abdumalik Davlatov, Sitora Janobilova, Amu Therwath, Massoud Mirshahi

**Affiliations:** Department of Plastic, Reconstructive Microsurgery and Regenerative Medicine, Avicenna Tajik State Medical University, Dushanbe, Tajikistan; Sorbonne Cité Paris, INSERM U965, University Paris 7, Lariboisiere Hospital, 41 bd de la Chapelle, 75010 Paris, France

**Keywords:** Asthma, Asphyxias, Laryngeal nerve, Surgical resection

## Abstract

**Background:**

Management of asthma in chronically affected patients is a serious health problem. Our aim was to show that surgical treatment of chronic bronchial asthma by unilateral resection of the internal branch of the superior laryngeal nerve (ib-SLN) is an adequateand lasting remedial response.

**Patients and methods:**

In a retrospective study, 41 (26 male and 15 female) patients with bronchial chronic asthma were treated surgically during the period between 2005 and 2013. It consisted of a unilateral resection of the ib-SLN under optical zoom, on patients placed in supinator position. 35 patients (24 male and 11 female) who were un-operated were included as a control.

**Results:**

In all patients, medication was reduced progressively. When the results were compared with the control group, it was seen that in 26 % of the patients, both forced expiratory volume (FEV) and peak expiratory flow (PEF) increased significantly (*p <05*) and only modestly in 53.6 % of patients (FEV, *p* <05 and PEF, *p* <05). In the remaining 20 % of patients, these parameters remained however unchanged. Overall, in 80 % of patients unilateral resection of the ib-SLN gave satisfactory results because it shortened the intervals and duration of asthmatic attacks, rendering thereby a reduction in medication.

**Conclusion:**

This minimal-invasive method helped prevent/cure asphyxias in chronic bronchial asthma without affecting cough reflex,respiratory control and phonation and it helped patients avoid severe crisis. This approach is of interest for patients with severe and/or uncontrolled bronchial asthma in settings with limited access to drug treatment.

## Background

Inspite of major achievements in pharmacotherapy and improvements in treatment protocols, management of chronic bronchial asthma (CBA) remains an important problem in modern medicine. Recent decades have registered a constant increase in CBA and it seems that at present 5 % of the global population suffers from it [1, 2]. TheAmerican Lung Association in 2012 reported that there were about 300 million personsin the world suffering from asthma. Two hundred fifty thousand people are known to die each year from severe asthmatic crisis [3].

Currently, CBA is treated by corticosteroids and adrenomimetic drugs. Unfortunately, all medical protocols still remain expensive, lengthy, providing only temporary relief and beset with several side effects [4]. Often at the moment an asthmatic crisis occurs, none of these drugs are necessarily at hand or in possession of the patients. This could be the situation in far flung remote areas in different parts of the world. The surgical method is an alternative approach for the treatment of severe bronchial asthma and has been commented critically by several authors [5–10]. Methods of surgical treatment of CBA are classified as: 1) tissue-therapy including glomectomy, pulmonary roots denervation, ganglionectomy and vagotomy as first generation - 2) intervention in vegetative nervous system such as implantation of neurostimulators of sinocarotid, vagus and diaphragmal nerves, sympathic trunks as second generation and 3) implants of programmed microchips as third generation therapy [11, 12]. Resection of the internal branch of the superior laryngeal nerve was proposed by Ulmer and Schlenkhoff as a surgical treatment of CBA in 1980 [13]. This was a novel approach and a turning point in the clinical handling of CBA. However, this innovative approach was progressively abandoned in the face of certain post-operative problems due to the invasive surgery involved.

In this study, a minimal-invasive surgical treatment of chronic bronchial asthma by unilateral resection of the internal branch of the superior laryngeal nerve is proposed.

## Methods

During the period of 2005 through 2013, the Department of Plastic and Reconstructive Microsurgery treated 41 patients with bronchial asthma (16 females and 25 males) with age ranging from 21–74 years (42.7 ± 13.4 years). Of these seven patients had been cigarette smokers. Patients targeted for treatment were those; i) suffering from chronic asthma of average duration 12.6 ± 10 years (ranging from 1.5–39 years), ii) with attack-frequency varying from 1 tо 4 times a week and, iii) duration of hospitalization more than 5 days. Characteristics for operated patients are resumed in Table 1A.

**Table 1:** A and B: characteristics of operated patients (1A) and non-operated patients as control group (1B)

A			
MALE	FEMALE	AGE OF PATIENTS M ± SD	DURATION OF ASTHMA/YEAR M ± SD
26	15	38,5 ± 13,8	13,2 ± 9,4
B			
MALE	FEMALE	AGE OF PATIENTSM ± SD	DURATION OF ASTHMA/YEAR M ± SD
24	11	39,6 ± 13,4	13,7 ± 9,2

A second group of 35 patients with the same criteria of chronic bronchial asthma (attack-frequency and duration of hospitalization), aged 16 to 67 years (11 males and 24 females) with disease duration of 13.5 ± 3.6 years were given only traditional therapy (drug therapy without surgery) and were considered as the control group (Table 1B).

All patients were under observation during the post operation period which ranged from 6–96 months with mean of 43.1 ± 32.3 months and a median of 39 [17-78] months for operated group and from 6 to 96 months with mean of 35,7 ± 25.1 months for control.

Patients admitted to the department for the surgical treatment of asthma, had pre- and postoperative clinical examinations, which included laboratory tests (spirometry such as forced expiratory volume (FEV1) and peak expiratory flow (PEF), ECG and R-graph of the chest), and consultation with apneumologist. Written consent was obtained from all patients willing to participate in the study. The ethics committees of the Tajik Health Ministry gave its approval of the procedures to be followed and for undertaking this study.

Surgery was performed under local anesthesia, in combination with leptoanalgesia with monitoring of the cardiovascular and respiratory systems.

### Position

**Fig. 1:**
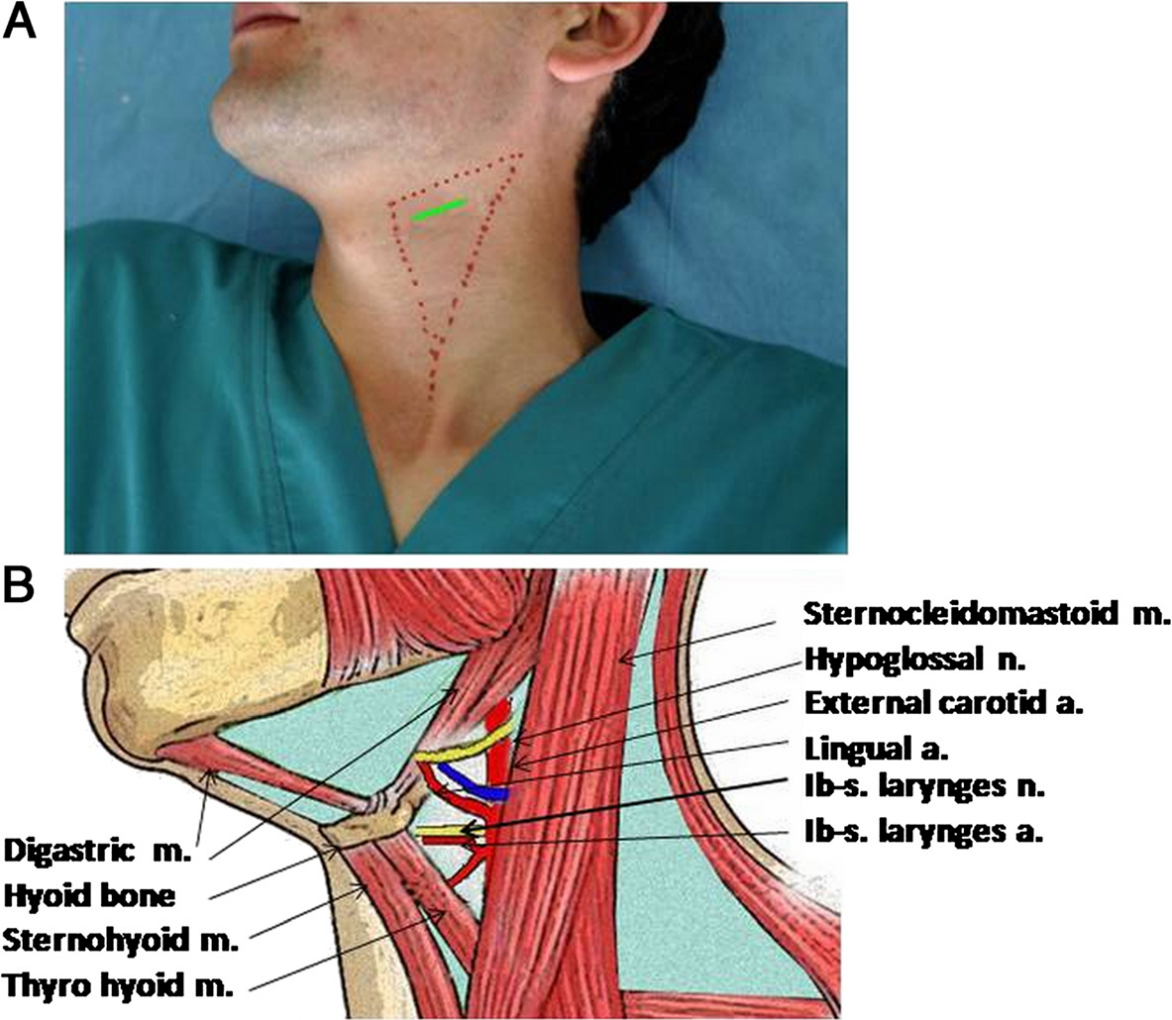
Position and anatomic description of *trigonumcaroticum*area*:* **a** The position of oblique-transverse approach (greenline) to anatomic projections on the neck. **b** The position of inner branch of superior laryngeal nerve and artery

Prior to the operation, the incision areas were carefully marked taking into account the comfort for operation and the esthetic demands. Briefly, it was necessary to demarcate on *trigonum caroticum* and ascertain the superior limit of the posterior digastric muscle, on the lateral by sternocleidomastoid muscle and from medial by superior belly of omohyoide and thyrohyoid muscles. A final marking for the actual incision and subsequent microsurgery was made as displayed in Fig. 1.

### Anesthesia

The operation was performed under local anaesthesia administered by infiltration of a solution of 1 % lidocaine with epinephrine (1:100,000) from outside to inside, in layers as well as on the lateral sides of the incision areas to block all cutaneous sensory nerve branches. This local anesthesia lasted from one to one and a half hours, which was enough time for the surgical act.

### Surgical methods

**Fig. 2:**
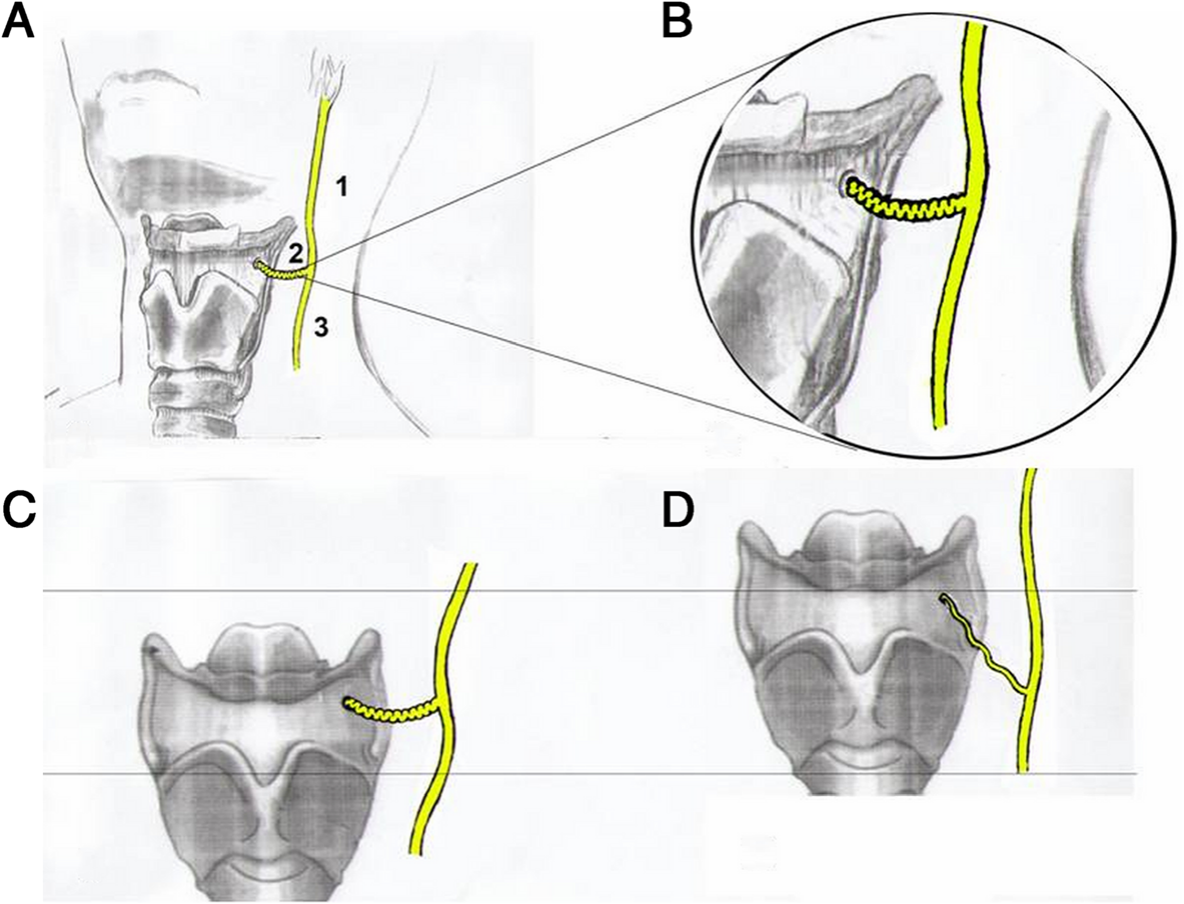
**a** Anatomic position of inner branch of superior laryngeal nerve (1), ramus intern (2), ramus extern (3). **b** use of optic magnification × 2.5 for visualization of inner branch of superior laryngeal nerve. **c** Internal branch of Laryngeal superior nerve (curved form) penetrates the side of the thyro-hyoid membrane into the cavity of the larynx. **d** In the act of swallowing, when the whole complex anatomical rises nerve just straightened

The ib-SLN is vulnerable during surgical interventions of the anterior cervical region and care must be taken to maintain it during the procedure. It should be noted that the surgery must strictly be performed under optical magnification (× 2.5), for clear discernment and visual ease. Briefly, an incision of 3–4 cm on predetermined and marked line (Fig. 1) was made. Тhus, the zone of search waslimitedto an area of 2.5 сmin diameter. Internal branch ofthe superior laryngeal nerve was evidenced immediately behind the external carotid and superior laryngealarteries, and sometimes 1–3 mm above (Fig. 2a). Ib-SLN cannot be confused with other structures, due to severe tortuosity (Fig. 2a, b) and iridescent white ivory colour. At the demand of the patient to the act of swallowing, there is increased mobility and unfolding of the nerve trunk. In addition this nerves in the act of swallowing, moves in rhythm with the thyroid cartilage and the thyrohyoid membrane (Fig. 2c, d). Once the ib-SLN was clearly identified, the 0.5–1 cm resection from the trunk of the internal branch of the superior laryngeal nervewas performed andoperative incisions were closed by applying Steri-strip or Proxi-strip. Skin stitches were removed 5–7 day after the operation. The criteria for evaluation were: 1) subjective feeling of the patient of well-being, 2) spirometry after surgery such as forced expiratory volume (FEV1) and peak expiratory flow (PEF) evaluation.

### Statistical analyses

All values reported are the average ± SEM. Statistical significance was determined using the GraphPad Prism 6.0 software (Kruskal-Wallis test).

## Results and discussion

The internal branch of the superior laryngeal nerve (ib-SLN) is composed of mostly sensory fibers for the major part of the laryngeal mucosa and the laryngopharynx and protects the respiratory tract by mobilization of the glottis closure reflex during swallowing, coughing, and vomiting [14, 15]. The cough reflex protects the laryngeal aditus from aspiration of secretions and diminishes the chances of aspiration-pneumonia [16]. Furthermore, bilateral damage of theib-SLN could lead to phonation disorders [17] and disorders of respiratory control [18].

The post-operative period was uneventful and recovery was, smooth. In general, patients requiring no further attention were discharged either the same day or after an overnight stay. Further care and follow up treatment was givenat the out-patient sector of the hospital.

The post-operative evaluation of the results is presented in Table 2. In all patients, medication was reduced progressively. When the results were compared with the control group, in 26 % of the patients, both FEV and PEF increased significantly (*p <05*) while in 53.6 % of patients only modestly (FEV1, *p* <05 and PEF, *p* <05) increased. In parallel, these parameters, in 20 % of the patients, remained unchanged (Table 2). Overall, in 80 % of patients unilateral resection of the ib-SLN gave satisfactory results because it shortened the intervals and duration of asthmatic attacks rendering thereby a reduction in medication.

**Table 2:** Objective information from spirometry in pre- and post- operated patients

	Pre-operation		Post-operation	
Cases	FEV1	PEF	FEV1	PEF
Good results 11 (26 %)	55 ± 10 %	42 ± 9 %	81 ± 4 % *(p <05)*	71 ± 4 % *(p <05)*
Satisfactory 22 (53.6 %)	42.5 ± 8 %	35 ± 5 %	59 ± 6 % *(p <05)*	41 ± 6 % *(p <05)*
Ineffective 8 (20 %)	35 ± 6 %	26 ± 9 %	No any changes	
Hematoma			Not occurred	
Aspiration			Not occurred	
loss of cough reflex			Not occurred	
loss of phonation			Not occurred	

FEV1: forced expiratory volume, PEF: peak expiratory flow

At the moment of the operation, 31 patients were corticosteroid dependent. After the operation, for 11 patients all drugs administration was stopped completely and in eight cases corticosteroids were also dropped. In case of 22 patients, alternative drug therapy (β2 adrenergic antagonist, salbutamol) was effective with progressive dose reduction. In all operated patients, the duration of hospitalization as well as the frequencies of asthma attacks was decreased. The clinical state of operated patients, compared with the controls, is presented in Table 3.

**Table 3:** Medical situation of operated patients compared with control. 31 of 41 patients were used corticosteroids

	Operated patients	Control Only medical treatment
Stopped taking corticosteroids	19/31patients (61 %)	0
Shortening of reception salbutamol	22/41patients, 1–3 times/week	all control patients, 3–5 times/day
Duration of hospitalization	1–3 days	5–10 days
decrease of asthma attack	1–2 per month	1–3 in the week

In the cases reported here the use of precision engineering and optical zoom, minimal incision, accurate identification of nerve and minimally injured tissue, made it possible to avoid unwanted postoperative complications as reported by Nishino T [19]. It should be noted that in other studies reported by Ulmer WT [14] and Feofilov GL [11], the authors did not follow the rigors of the approach described by us which may explain, the occurrence in their cases, post-operative complications such as hematoma, sublingual paralysis and even death.

Overall in 20 % of patients with chronic disease (above 20 years of bronchial asthma) and elderly patients with concomitant complications in the lungs (pulmonary fibrosis, emphysema, chronic inflammatory diseases) surgical treatment did not improve the affection, which from a medical point of view was not surprising.

Undoubtedly, resection of the ib-SLN alleviated the suffering endured by the asthmatic patients. After the surgery the frequency of treatment by salbutamol, duration of hospitalization and asthma attacks were significantlyreduced.

The ib-SLN conveys mostly sensory fibers for the major part of laryngopharynx and protects the respiratory tract by mobilization of the glottis closure reflex [20]. Furthermore, in this study, unilateral resection of ib-SLN had not affected cough reflex, respiratory control, and phonation nor induced any hematoma in the patients.

It is worth noting that in the works of Schlenkhoff D [14] (171 patients) as well as in that of Melnikov V.M. [21] (51 patients) in the long-term results (20 years of observations), they did not report any postoperative inflammation or mucosal denervation injury. We also want to emphasize that in our case (41 patients) during the 96 months of observation, there were no case with postoperative inflammation of the mucous membrane or contralateral nerve injury. Injury to the unilateral SLN does not cause prompt, noticeable functional impairment of the vocal cords. The dual innervation of laryngeal mucosa from the contralateral SLN prevents loss of laryngeal sensation [22]. In our work, nerve resection was performed in all 41 cases with only from one side (unilateral).

Here, we modified the method of crossing the ib-SLN that was previously suggested by D. Schlenkhoff. This author performed the resection of the internal branch of laryngeal nerve by the traditional ‘open sky’ way without resorting to precision techniques as we did. It was not surprising, as admitted by these authors, that there were several side effects involving the facial nerve, the hypoglossal nerve and the external branch of superior laryngeal nerve. Their technique was lethal in 6 % of the patients [14].

Our objective was to avoid complications faced by the earlier authors, obtain equally satisfactory results while causing the least trauma to the patients. Our approach was minimum-invasive. The incision was well targeted and was no more than 3 to 4 cm long. The internal branch of superior laryngeal nerve was identified using the optical zoom for magnification and unilateral resection was performed on the ib-SLN following well-rehearsed precision technique and the use of *microtoolkit*. The patients were placed in supinator position. This way we managed to achieve a minimum invasiveness and reduce the duration of the operation to 20 to 30 min.

There are other approaches (e.g. thermoplasty) which might be an alternative with still less risk involved in order to help severe asthmatics [23], for review). One may mention that nerve resection may be performed in several surgical designs such as a rhizotomy procedure, sectioning the intermediate nerve for the treatment of laryngeal neuralgia [24] and denervation of renal sympathetic nerve for treating resistant hypertension [25].

Here, our approach reduced the hospital stay to 1 day or to a maximum of 2 days. The method was applicable with a minimum possible financial burden, both for pre-operative preparation of the patients or for post-operative care. Thus the operation once standardized and perfected using video fluoroscopy combined with manometer for all œsophagien syndromes [26], can be performed in any simple but adequate medical set-up without resorting to general anesthesia.

Further studies are needed for understanding the mechanism of allergen tolerances by the immune system after resection of the laryngeal nerve.

By introducing changes and innovation, we were able to improve on the surgical approach to treatment of chronic bronchial asthma and propose its re-introduction in clinical practice. This method can be adapted in other situations mimicking asthmatic syndrome resulting from mechanical invasion of branchial airways [27].

## Conclusion

In conclusion, our experience shows that ib-SLN resection is an easy and practically safe method for obtaining a permanent relief for a large number of patients from chronic bronchial asthma and asphyxia resulting from severe crisis.

## Abbreviations

EVC: Expiratory Vital Capacity
FEVC: Forced Expiratory Vital Capacity, FEVC1 (in one second)
IVC: Inspiration Vital Capacity
MEF: Maximum Expiratory Flow
PEF: Peak Expiratory Flow
